# *Moraxella osloensis* Isolated from the Intraoperative Field After Reverse Total Shoulder Arthroplasty

**DOI:** 10.3390/microorganisms13122699

**Published:** 2025-11-26

**Authors:** Enrico Bellato, Fabio Longo, Francesca Menotti, Claudia Pagano, Antonio Curtoni, Alessandro Bondi, Filippo Castoldi, Giuliana Banche, Valeria Allizond

**Affiliations:** 1 Department of Surgical Sciences, University of Torino, 10126 Turin, Italy; enrico.bellato@unito.it; 2 Department of Public Health and Pediatrics, University of Torino, 10126 Turin, Italyfrancesca.menotti@unito.it (F.M.); antonio.curtoni@unito.it (A.C.); alessandro.bondi@unito.it (A.B.); valeria.allizond@unito.it (V.A.)

**Keywords:** *Moraxella osloensis*, intraoperative swabs, operating room contamination, susceptibility pattern, biofilm formation

## Abstract

*Moraxella osloensis* is an infrequently reported component of the human skin microbiota, but it has recently been recognized as a potential source of intraoperative contamination. Its pathogenic role remains poorly defined, particularly in shoulder arthroplasty. This study describes the recovery and characterization of *M. osloensis* from intraoperative periprosthetic tissue samples collected immediately after reverse total shoulder arthroplasty in five patients. All isolates exhibited low colony counts (10–50 CFU/mL), were uniformly susceptible to the antimicrobial agents tested, and did not produce β-lactamases. Biofilm formation—an important virulence determinant in periprosthetic joint infections—was detected in two of the five isolates. Clinically, no patient developed postoperative infection within 12 months, and only one experienced a transient superficial wound-healing delay, which resolved with a short administration of oral antibiotics. These findings indicate that *M. osloensis* may be present in the operative field despite stringent skin preparation and aseptic protocols, likely reflecting endogenous colonization rather than environmental contamination. Although its clinical impact appears limited in this context, the bacteria’s biofilm-forming potential and underrecognized presence in the operating room underscore the importance of continued surveillance and careful interpretation when isolated from surgical specimens.

## 1. Introduction

During reverse total shoulder arthroplasty (RTSA), several adverse events can occur, especially prosthetic joint infections (PJIs) and surgical site infections (SSIs) [1,2,3,4,5,6,7,8,9,10]. The pathogens most commonly recovered from shoulder PJIs and SSIs are *Cutibacterium acnes* and coagulase-negative staphylococci (CoNS), which account for nearly 42% and 33%, respectively, of all positive cases [11]. In addition, *Staphylococcus aureus* and Gram-negative bacteria, such as *Escherichia coli*, can also be isolated [12]. In addition to aseptic surgical techniques as well as antibiotic prophylaxis and skin disinfection protocols, some fastidious and uncommon microorganisms can also contaminate the surgical area [13,14,15,16]. In fact, the operating room air and personnel present can transport different microorganisms to determine not only contamination in the area but also infection in patients undergoing surgery. Among them, *Moraxella osloensis*, a Gram-negative and aerobic coccobacillus, normally resides in human skin, mucus membranes and the respiratory tract [17]. It has recently emerged as a bacterium present in the operating room and is derived from surgical devices [12,18]. Moreover, it has been demonstrated to cause human diseases such as unusual osteomyelitis in healthy subjects [17] and joint septic arthritis in adult patients [19,20,21].

Here, we describe five cases of *M. osloensis* recovery from intraoperative swabs collected from patients who underwent RTSA at A.O.U. San Luigi Gonzaga Hospital, Orbassano (Italy), immediately after implantation. The *M. osloensis* strains were further characterized for their antimicrobial susceptibility pattern and for their ability to produce biofilms, which are the main recognized virulence factors. All the patients described here were followed up at 7 and 14 days and at 2, 6 and 12 months postoperatively via the subjective shoulder value (SSV) and the Oxford shoulder score (OSS). Shoulder X-rays were collected at 6 and 12 months postoperatively [22,23].

## 2. Materials and Methods

### Microbiological Analysis

Immediately before surgical field preparation, the patient’s shoulder and axilla were scrubbed with 7.5% povidone-iodine, rinsed with water, and then prepared with 4% chlorhexidine gluconate [13]. Sterile, non–iodine-impregnated adhesive drapes were applied. Following both skin (deltopectoral approach) and subcutaneous incisions, the scalpel blade was replaced [24]. At the end of RTSA, a periprosthetic tissue sample was collected, placed in Amies medium (CultureSwab, Becton Dickinson, BD, Milan, Italy), and delivered within 1 h, for microbiological investigation. A negative control represented by a swab placed in the surgical field without sampling any internal area was also collected.

At the Bacteriology and Mycology Laboratory, Department of Public Health and Pediatrics, University of Torino, the specimens were sonicated and vortexed, and 100 µL was seeded on Columbia agar with 5% sheep blood (BD, Italy) for aerobic bacteria, on Schaedler Agar with 5% blood (BD, Italy) for anaerobic bacteria, and on Sabouraud dextrose agar (Biokar Diagnostics, Alonne, France) for fungi. The number of Colony-Forming Units (CFUs)/mL was recorded after the incubation at 37 °C for up to 10 days under aerobic conditions for aerobic bacteria and fungi and for up to 21 days under strictly anaerobic conditions within an anaerobic system (Gaspak EZ anaerobe pouch system kit, BD, Italy) for anaerobic bacteria [13]. A preliminary identification was performed through Gram stain, catalase and oxidase reactions. Thereafter, the isolates were definitively identified by mass spectrometry via matrix-assisted laser desorption/ionization time-of-flight (MALDI-TOF) technology (Bruker Daltonics GmbH, Bremen, Germany), with a score ≥ 2 deemed suitable [25].

To determine their antimicrobial patterns, the strains were subjected to antimicrobial susceptibility testing for uncommon aerobic microorganisms lacking species-related breakpoints via the E-test method (bioMérieux Italia S.p.A, Bagno a Ripoli, Firenze, Italy), according to European Committee on Antimicrobial Susceptibility Testing (EUCAST) guidelines. A 0.5 McFarland density inoculum, corresponding to ~1–2 × 10^8^ CFUs/mL, was plated on Mueller Hinton fastidious agar supplemented with 5% horse blood + 20 mg/l β-NAD (BD), incubated under aerobic conditions at 37 °C, and evaluated after 18–24 h. The following antimicrobial drugs were assayed: ampicillin (0.016–256 µg/mL), amoxicillin/clavulanic acid (0.016–256 µg/mL), cefotaxime (0.002–32 µg/mL), ciprofloxacin (0.002–32 µg/mL), cefepime (0.016–256 µg/mL), erythromycin (0.016–256 µg/mL), tetracycline (0.016–256 µg/mL), and trimethoprim-sulfamethoxazole (0.002–32 µg/mL) (bioMérieux Italia S.p.A., Italy). Owing to the lack of specific breakpoints from the EUCAST, those for bacteria lacking breakpoints in standard EUCAST breakpoint tables were used (https://www.eucast.org/fileadmin/src/media/PDFs/EUCAST_files/Guidance_documents/When_there_are_no_breakpoints_2024-09-03.pdf; accessed on 3 March 2025).

The β-Lactamase production was also evaluated by using β-lactamase test purchased from Liofilchem^®^ s.r.l. (Roseto degli Abruzzi, Teramo, Italy).

Finally, the ability of the isolates to produce biofilms was characterized by Tissue Culture Plate (TCP) since it is the most widely used method thus being considered the gold standard for detection of biofilm formation [26]. Briefly, 200 µL of a 0.5 McFarland suspension of each strain in trypticase soy broth (TSB; Merck KGaA, Darmstadt, Germany) supplemented with 5% glucose was inoculated in a 96-well microplate and incubated for 24 h at 37 °C. Wells containing only TSB were used as negative controls. Thereafter, once the planktonic cells were removed, three washes with distilled water were performed. Next, 200 µL of methanol (Carlo Erba Reagents S.r.l., Cornaredo, Milan, Italy) was added for 15 min to fix the adhered bacteria, followed by 15 min of staining with 200 µL of 1% crystal violet (Merck KGaA). Then, three more washes were carried out, and once the plate was dry, 200 µL of 95% ethanol (Carlo Erba Reagents S.r.l.) was added to each well. Finally, the optical density (OD) at 595 nm was measured via a microplate reader (VICTOR3TM, PerkinElmer, Waltham, MA, USA). To categorize the different levels of adhesion (i.e., to assess the attachment and aggregate formation capabilities necessary for biofilm formation) an OD cut-off value (ODc) was calculated by adding three standard deviations to the negative control mean OD. The strains were classified for their biofilm-producing ability, as shown in Table 1.

## 3. Results

### 3.1. Case Description

Patient 1 was a 68-year-old non-smoking woman (75 kg weight, 1.60 m height, BMI 29.30 kg/m^2^) with chronic atrial fibrillation. The patient, affected by shoulder osteoarthritis, was treated with cortisone injections more than 12 months before surgery. Preoperative antibiotic prophylaxis consisted of cefazolin 2 g, and the surgery lasted 84 min. The patient did not experience either intra- or postoperative complications and did not experience complications during the follow-up period. No radiographic signs of mobilization or infection were observed. At the final follow-up, the SSV was 90%, and the OSS was 47.

Patient 2 was a 74-year-old non-smoking man (60 kg weight, 1.70 m height, BMI 20.76 kg/m^2^) with dyslipidemia. The patient was affected by a massive irreparable rotator cuff tear. He underwent cortisone injection therapy more than 12 months before surgery. After antibiotic prophylaxis with cefazolin, the patient underwent RTSA, which lasted 92 min. No intraoperative complications were observed. A mild hematoma on the anterior side of the arm was observed at 7 and 15 days postoperatively. The surgical wound exhibited a delay in the healing process, and at 2 months after surgery, the patient presented with a small (5 mm) degree of wound dehiscence without drainage and mild redness. The patient never reported fever. A culture swab from the surgical wound was obtained, with negative results. Blood tests were negative for infection (WBC 7000/microl, CRP 0.1 mg/L, ESR 2 mm/h, and procalcitonin 0.02 ng/mL), and the patient was empirically treated with amoxicillin clavulanate for 7 days with success. At 12 months postoperatively, the patient was pain free, and no local or radiographic signs were further observed. The SSV was 95%, and the OSS was 45.

Patient 3 was an 84-year-old non-smoking man (78 kg weight, 1.75 m height, BMI 25.47 kg/m^2^) with dyslipidemia and hypertension as comorbidities. The patient, affected by cuff tear arthropathy, did not receive cortisone injections before surgery. He underwent antibiotic prophylaxis with 2 g cefazolin. The surgery lasted 94 min. No intraoperative complications occurred. At 7 days after surgery, a hematoma on the anterior side of the arm was observed, which completely resolved spontaneously in 15 days. At 12 months post-surgery, no radiographic signs of mobilization or infection were observed. The patient scored his shoulder 100% with the SSV and 48% with the OSS.

Patient 4 was a 79-year-old non-smoking woman (42 kg weight, 1.60 m height, BMI 16.41 kg/m^2^) with dyslipidemia and hypertension. The patient was diagnosed with cuff tear arthropathy and was not treated with cortisone injections. Antibiotic prophylaxis consisted of cefazolin 2 g, and RTSA was performed within 97 min. The patient did not experience either intra- or postoperative complications. At the latest follow-up, no radiographic signs of mobilization or infection were noted, and the SSV and OSS were 60% and 33, respectively.

Patient 5 was a 78-year-old non-smoking woman (72 kg weight, 1.62 m height, BMI 27.43 kg/m^2^) with dyslipidemia, hypertension, diabetes mellitus and dysthyroidism as comorbidities. The patient was affected by cuff tear arthropathy and did not receive cortisone injections during the 12 months before surgery. After antibiotic prophylaxis with 2 g cefazolin, the patient underwent RSTA (90 min). The patient did not experience either intra- or postoperative complications. Twelve months after surgery, no radiographic signs of mobilization or infection were observed. The patient scored her shoulder 98% using the SSV, and the OSS was 48.

### 3.2. Microbiological Findings

**Figure 1:**
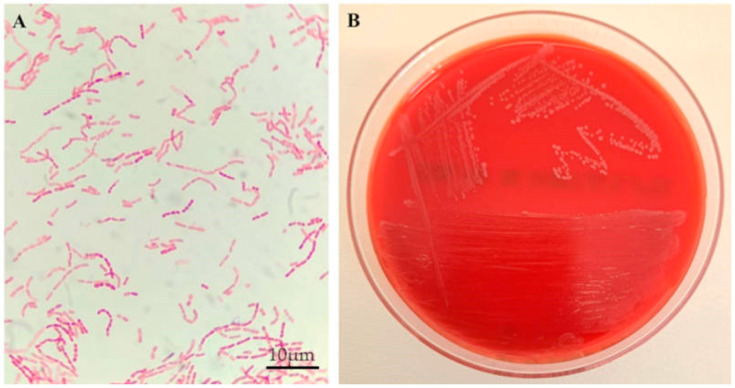
Representative images of *M. osloensis:* Gram stain (**A**) and pure subculture (**B**).

The cultures were grown under aerobic conditions for 2 days, at which point the positivity and load were recorded. Among the samples, Gram-negative, catalase- and oxidase-positive coccobacilli were observed, and MALDI-TOF identification revealed a strain of *M. osloensis* (>2 score) in all the samples. Figure 1 shows representative images of a Gram stain (Figure 1A) of an *M. osloensis* isolate and its subculture on Columbia agar with 5% sheep blood (Figure 1B). No additional microorganisms were detected on any of the agar media used for culture or in the negative controls.

**Figure 2:**
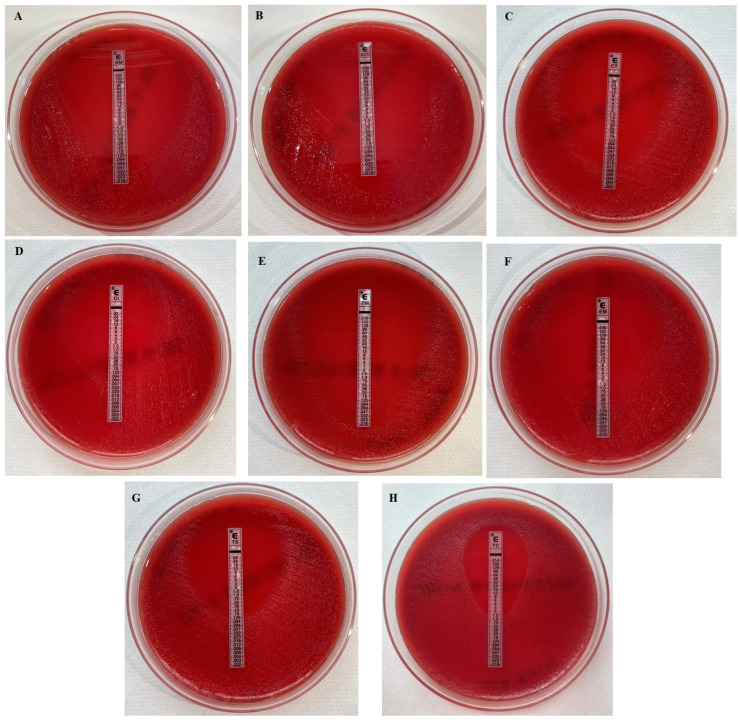
Representative image showing the susceptibility patterns of the *M. osloensis* isolates to different antimicrobial agents, as determined via the E-test: ampicillin (**A**), amoxicillin/clavulanic acid (**B**), cefotaxime (**C**), ciprofloxacin (**D**), cefepime (**E**), erythromycin (**F**), trimethoprim-sulfamethoxazole (**G**), and tetracyclines (**H**).

The microbial load of the *M. osloensis* strains isolated from the intraoperatory tissues samples was determined by counting the CFUs/mL. Specifically, 50 CFUs/mL, 20 CFUs/mL, 30 CFUs/mL, 10 CFUs/mL and 10 CFUs/mL were registered from Cases 1 to 5, respectively. The susceptibility patterns to different antimicrobials were obtained via the E-test method (Figure 2), and the results are detailed in Table 2. None of the strains were β-lactamase producers.

**Figure 3:**
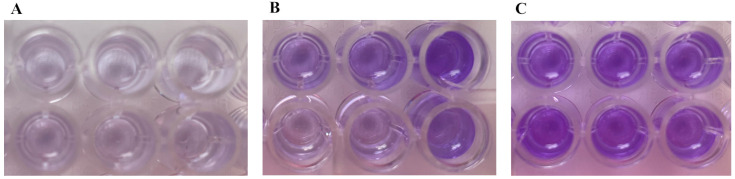
Representative images demonstrating the biofilm formation capability of the *M. osloensis* isolates by the crystal violet staining method: control (**A**), moderately (**B**) and strongly (**C**) adhered strains.

Finally, as shown in Figure 3, the biofilm formation of the *M. osloensis* strains revealed that 2 out of 5 (40%) strains formed biofilms with moderate or strong adhesion. In Table 3 a summary of the OD obtained and the strain characterization is showed.

## 4. Discussion

Joint replacement infection represents a very serious complication with significant consequences for both the patient’s health and the healthcare system due to high management costs [4,9]. In most cases, it is the result of intraoperative contamination, and the bacteria involved are primarily CoNS and, in the case of shoulder prostheses, *C. acnes* [27,28,29,30,31,32].

Notably, our study is the first to present cases of intraoperative contamination caused by *M. osloensis* during prosthetic shoulder surgery. This bacterium was identified with a load of approximately 24 CFUs/mL. Currently there is no universally accepted CFU threshold for defining significant intraoperative contamination for skin commensals such as *M. osloensis*; therefore, we present absolute counts and interpret them qualitatively. Moderate virulence features characterize the five strains: they displayed low MICs for all the assayed antibiotics, they were non-β-lactamase producers, and two of them were characterized by the ability to produce biofilms.

Literature reports revealed that this bacterium has been isolated from operating room environments. In particular, Gilat R. et al. (2020) highlighted the recovery of *M. osloensis* from protective lead garments—either inside or outside—that were worn in the operating room, identifying them as a potential source for microbial colonization and thus increasing the intraoperative infection risk for patients under surgery [12]. Moreover, loupes, headlights and battery packs in the operating room were found to be positive for *M. osloensis*, as were several types of surgical equipment [18]. Unfortunately, in none of these studies were the virulence factors of the *M. osloensis* isolates fully characterized. Here, *M. osloensis* was detected despite strict aseptic conditions and the double skin disinfection protocol applied [13]: it can be speculated that the endogenous skin microbiota of patients, rather than environmental sources such as air, drapes, or instruments, represents the most plausible origin of this bacterium detected intraoperatively.

Studies on the recovery of *M. osloensis* as a human pathogen are still rare. Alkhatib N.J. (2017) described a case of femur osteomyelitis in a healthy man due to *M. osloensis* with no evidence of infection unless intraoperative findings and magnetic resonance imaging were obtained. This infection was effectively treated with surgical debridement and intravenous third-generation cephalosporins [17]. In contrast, Quoilin M. et al. (2024) described septic arthritis of the cervical facet joint in a 66-year-old male caused by *M. osloensis*, but in this case, the patient suffered from numerous symptoms (i.e., pain, numbness and tingling episodes) [21]. Other cases of ankle or knee septic arthritis caused by *M. osloensis* have been identified [19,20]. Previously, other authors presented infections caused by this bacterium, ranging from central venous catheter infection, endocarditis, bacteremia, peritonitis, and genital infections to meningitis [17,33,34,35,36,37,38,39]. More recently, it was reported that *M. osloensis* was the etiological cause of blood infection in a 2-month-old girl: the isolate was generally susceptible to antibiotics, and the infection was successfully treated with 4 days of intravenous cefotaxime [40]. Importantly, in contrast to our findings, none of the aforementioned cases of *M. osloensis* recovery or subsequent infection occurred after prosthetic surgery. In fact, in our case, the 5 patients described, despite documented intraoperative contamination, did not develop infection (at least within the first 12 months after surgery), with only one case of suspected mild superficial infection without the identification of an etiological agent.

*M. osloensis* is considered a skin colonizer, and it is widely distributed in the skin microbiome [41,42,43]. Nasal skin samples were found to be resistant to antimicrobials, mainly β-lactams and aminoglycosides, when they carried the plasmid pNP7-1, but resistance to trimethoprim-sulfamethoxazole and amoxicillin/clavulanic acid of approximately 3% and 8%, respectively, was also highlighted, indicating that therapy with these agents should be discouraged, especially for Gram-negative bacteria [41,44,45,46]. In the present research, the five isolated strains were characterized by reduced virulence since they presented lower MICs than those above did

Another pathogenic factor associated with *M. osloensis* is its ability to produce biofilms that worsen infection outcomes in patients. Hadi Ghaffoori Kanaan M.H.G. et al. (2024) reported that 65.3% of the 19 strains isolated were strong biofilm producers [46]; notably, the strains here isolated were capable of producing biofilms, specifically 2 out of 5. Although literature reports in this field are still limited, considering the crucial pathophysiological role of biofilms in the development of periprosthetic infections [47], it is plausible that the ability of *M. osloensis* to form biofilms may enhance its persistence on surgical surfaces and prosthetic materials. It is also plausible that, similarly to other *Moraxella* species, this bacterium may employ immune evasion mechanisms [48]. Overall, these features could enable *M. osloensis* to pose a potential risk for postoperative infections despite its in vitro antibiotic susceptibility.

A limitation of this study is that microbiological analyses were not extended to other potential sources of contamination, such as the patients’ skin or environmental surfaces. As a result, the precise origin of *M. osloensis* cannot be definitively determined, although the patients’ own skin microbiota remains the most plausible source.

## 5. Conclusions

Although *M. osloensis* is rarely reported as a pathogen, it can also be found in the environment and within the operating room. In the present study, we report the isolation of five *M. osloensis* strains from intraoperative field tissues obtained from patients who did not develop postoperative infection. We believe that this microorganism is likely underdiagnosed due to its challenging culture requirements, which may lead to an underestimation of its actual pathogenic potential. Furthermore, its antimicrobial resistance profile and ability to form biofilms should be carefully monitored in surgical settings, as these features may contribute to a broad spectrum of infections, particularly in immunocompromised individuals.

Finally, in clinical cases where a definitive diagnosis of periprosthetic infection caused by common pathogens cannot be established and *M. osloensis* is isolated, its potential involvement in the infectious process should not be excluded but rather interpreted in close correlation with the patient’s clinical presentation.

## Figures and Tables

**Table 1:** Classification of strains for their ability to produce biofilms.

OD Value	Adhesion	Biofilm Production
OD ≤ ODc	Not adherent	Biofilm not producer
ODc < OD ≤ 2xODc	Weakly adherent	
2xODc < OD ≤ 4xODc	Moderately adherent	Biofilm producer
4xODc < OD	Strongly adherent	

Abbreviations: optical density, OD; optical density cut-off value, ODc.

**Table 2:** Summary of the distribution of minimum inhibitory concentrations (MICs), expressed as µg/mL, for the five *M. osloensis* isolates as revealed by the E-test methods.

Strain	Antimicrobial Drugs (MICs as µg/mL)							
	AMP	AMC	CFT	CIP	CFP	ERY	TET	TMP/SMX
1	0.023	≤0.016	0.004	0.032	≤0.016	0.38	1.5	0.064
2	0.032	≤0.016	0.004	0.19	≤0.016	0.75	1.5	0.125
3	0.19	≤0.016	0.016	0.094	0.032	0.38	0.75	0.094
4	0.094	≤0.016	0.032	0.125	≤0.016	0.75	0.75	0.19
5	0.094	≤0.016	0.012	0.016	≤0.016	0.125	1.5	0.125

Abbreviations: amoxicillin/clavulanic acid (AMC), ampicillin (AMP), cefepime (CFP), cefotaxime (CFT), ciprofloxacin (CIP), erythromycin (ERY), tetracyclines (TETs), and trimethoprim-sulfamethoxazole (TMP/SMX).

**Table 3:** *M. osloensis* strains ODs and ability to produce biofilms.

Strain	OD (Mean ± SD)	Biofilm Production
1	0.169 ± 0.047	Not detected
2	0.220 ± 0.149	Not detected
3	0.199 ± 0.121	Not detected
4	0.324 ± 0.166	Detected
5	0.546 ± 0.075	Detected

Abbreviations: optical density, OD; standard deviation, SD.

## Data Availability

The original contributions presented in this study are included in the article. Further inquiries can be directed to the corresponding authors.

## References

[1] Bonnevialle N., Dauzères F., Toulemonde J., Elia F., Laffosse J.-M., Mansat P. (2017). Periprosthetic Shoulder Infection: An Overview. EFORT Open Rev..

[2] Duvall G., Kaveeshwar S., Sood A., Klein A., Williams K., Kolakowski L., Lai J., Enobun B., Hasan S.A., Henn R.F. (2020). Benzoyl Peroxide Use Transiently Decreases Cutibacterium Acnes Load on the Shoulder. J. Shoulder Elb. Surg..

[3] Hsu J.E., Matsen F.A., Whitson A.J., Bumgarner R.E. (2020). Cutibacterium Subtype Distribution on the Skin of Primary and Revision Shoulder Arthroplasty Patients. J. Shoulder Elb. Surg..

[4] Hsu J.E., Neradilek M.B., Russ S.M., Matsen F.A. (2018). Preoperative Skin Cultures Are Predictive of Propionibacterium Load in Deep Cultures Obtained at Revision Shoulder Arthroplasty. J. Shoulder Elb. Surg..

[5] Kadler B.K., Mehta S.S., Funk L. (2015). Propionibacterium Acnes Infection after Shoulder Surgery. Int. J. Shoulder Surg..

[6] Lo E.Y., Ouseph A., Badejo M., Lund J., Bettacchi C., Garofalo R., Krishnan S.G. (2023). Success of Staged Revision Reverse Total Shoulder Arthroplasty in Eradication of Periprosthetic Joint Infection. J. Shoulder Elb. Surg..

[7] Lynch B.C., Swanson D.R., Marmor W.A., Gibb B., Komatsu D.E., Wang E.D. (2022). The Relationship between Bacterial Load and Initial Run Time of a Surgical Helmet. J. Shoulder Elb. Arthroplast..

[8] Matsen F.A., Whitson A.J., Hsu J.E. (2020). While Home Chlorhexidine Washes Prior to Shoulder Surgery Lower Skin Loads of Most Bacteria, They Are Not Effective against Cutibacterium (Propionibacterium). Int. Orthop. (SICOT).

[9] Matsen F.A., Whitson A.J., Pottinger P.S., Neradilek M.B., Hsu J.E. (2020). Cutaneous Microbiology of Patients Having Primary Shoulder Arthroplasty. J. Shoulder Elb. Surg..

[10] Saad M.A., Moverman M.A., Da Silva A.Z., Chalmers P.N. (2024). Preventing Infections in Reverse Shoulder Arthroplasty. Curr. Rev. Musculoskelet. Med..

[11] Hansen P.Y., Fomunung C., Lavin A., Daji A., Jackson G.R., Sabesan V.J. (2024). Outcomes Following Revision Reverse Shoulder Arthroplasty for Infection. J. Shoulder Elb. Surg..

[12] Gilat R., Mitchnik I., Beit Ner E., Shohat N., Tamir E., Weil Y.A., Lazarovitch T., Agar G. (2020). Bacterial Contamination of Protective Lead Garments in an Operating Room Setting. J. Infect. Prev..

[13] Blonna D., Allizond V., Bellato E., Banche G., Cuffini A.M., Castoldi F., Rossi R. (2018). Single versus Double Skin Preparation for Infection Prevention in Proximal Humeral Fracture Surgery. BioMed Res. Int..

[14] van Diek F.M., Pruijn N., Spijkers K.M., Mulder B., Kosse N.M., Dorrestijn O. (2020). The Presence of Cutibacterium Acnes on the Skin of the Shoulder after the Use of Benzoyl Peroxide: A Placebo-Controlled, Double-Blinded, Randomized Trial. J. Shoulder Elb. Surg..

[15] Panther E.J., Hao K.A., Wright J.O., Schoch J.J., Ritter A.S., King J.J., Wright T.W., Schoch B.S. (2022). Techniques for Decreasing Bacterial Load for Open Shoulder Surgery. JBJS Rev..

[16] Symonds T., Grant A., Doma K., Hinton D., Wilkinson M., Morse L. (2022). The Efficacy of Topical Preparations in Reducing the Incidence of Cutibacterium Acnes at the Start and Conclusion of Total Shoulder Arthroplasty: A Randomized Controlled Trial. J. Shoulder Elb. Surg..

[17] Alkhatib N.J., Younis M.H., Alobaidi A.S., Shaath N.M. (2017). An Unusual Osteomyelitis Caused by *Moraxella osloensis*: A Case Report. Int. J. Surg. Case Rep..

[18] Purcell C., Moolman N., MacKay C., Hatchette T.F., Trites J., Taylor S.M., Rigby M.H., Hart R.D. (2019). Bacterial Contamination of Surgical Loupes and Headlights. J. Laryngol. Otol..

[19] Banks L.N., Kurdy N.M., Hassan I., Aster A.S. (2007). Septic Arthritis of the Ankle Due to *Moraxella osloensis*. Foot Ankle Surg..

[20] Pratibha D., Lakshmi D., Gomathy D., Kumar P., Vanishree Y.M. (2017). *Moraxella osloensis*: Septic Arthritis. Saudi J. Pathol. Microbiol..

[21] Quoilin M., Vu P.D., Bansal V., Chen J.W. (2024). Septic Arthritis of the Cervical Facet Joint: Clinical Report and Review of the Literature. Pain. Pract..

[22] Dawson J., Fitzpatrick R., Carr A. (1996). Questionnaire on the Perception of Patients about Shoulder Surgery. J. Bone Jt. Surg. Br. Vol..

[23] Gilbart M.K., Gerber C. (2007). Comparison of the Subjective Shoulder Value and the Constant Score. J. Shoulder Elb. Surg..

[24] Levy B.J., Grimm N.L., Jimenez A.E., Shea K.P., Mazzocca A.D. (2021). Is There Value in the Routine Practice of Discarding the Incision Scalpel from the Surgical Field to Prevent Deep Wound Contamination with Cutibacterium Acnes?. J. Shoulder Elb. Surg..

[25] Koleri J., Petkar H.M., Husain A.A.M., Almaslamani M.A., Omrani A.S. (2022). *Moraxella osloensis* Bacteremia, a Case Series and Review of the Literature. IDCases.

[26] Christensen G.D., Simpson W.A., Younger J.J., Baddour L.M., Barrett F.F., Melton D.M. (1985). Adherence of coagulase-negative staphylococci to plastic tissue culture plates: A quantitative model for the adherence of staphylococci to medical devices. J. Clin. Microbiol..

[27] Maccioni C.B., Woodbridge A.B., Balestro J.-C.Y., Figtree M.C., Hudson B.J., Cass B., Young A.A. (2015). Low Rate of Propionibacterium Acnes in Arthritic Shoulders Undergoing Primary Total Shoulder Replacement Surgery Using a Strict Specimen Collection Technique. J. Shoulder Elb. Surg..

[28] Pauzenberger L., Grieb A., Hexel M., Laky B., Anderl W., Heuberer P. (2017). Infections Following Arthroscopic Rotator Cuff Repair: Incidence, Risk Factors, and Prophylaxis. Knee Surg. Sports Traumatol. Arthrosc..

[29] Matsen F.A., Whitson A., Hsu J.E. (2020). Preoperative Skin Cultures Predict Periprosthetic Infections in Revised Shoulder Arthroplasties: A Preliminary Report. JBJS Open Access.

[30] Hudek R., Brobeil A., Brüggemann H., Sommer F., Gattenlöhner S., Gohlke F. (2021). Cutibacterium Acnes Is an Intracellular and Intra-Articular Commensal of the Human Shoulder Joint. J. Shoulder Elb. Surg..

[31] Kajita Y., Iwahori Y., Harada Y., Takahashi R., Deie M. (2021). Incidence of Cutibacterium Acnes in Open Shoulder Surgery. Nagoya J. Med. Sci..

[32] Lemmens L., Geelen H., Depypere M., Munter P.D., Verhaegen F., Zimmerli W., Nijs S., Debeer P., Metsemakers W.-J. (2021). Management of Periprosthetic Infection after Reverse Shoulder Arthroplasty. J. Shoulder Elb. Surg..

[33] Buchman A.L., Pickett M.J., Mann L., Ament M.E. (1993). Central Venous Catheter Infection Caused by *Moraxella osloensis* in a Patient Receiving Home Parenteral Nutrition. Diagn. Microbiol. Infect. Dis..

[34] Shah S.S., Ruth A., Coffin S.E. (2000). Infection Due to *Moraxella osloensis*: Case Report and Review of the Literature. Clin. Infect. Dis..

[35] Vuori-Holopainen E., Salo E., Saxen H., Vaara M., Tarkka E., Peltola H. (2001). Clinical “Pneumococcal Pneumonia” Due to *Moraxella osloensis*: Case Report and a Review. Scand. J. Infect. Dis..

[36] Gómez-Camarasa C., Fernández-Parra J., Navarro-Marí J.M., Gutiérrez-Fernández J. (2018). *Moraxella osloensis* emerging infection. Visiting to genital infection. Rev. Esp. Quim..

[37] Maruyama Y., Shigemura T., Aoyama K., Nagano N., Nakazawa Y. (2018). Bacteremia Due to *Moraxella osloensis*: A Case Report and Literature Review. Braz. J. Infect. Dis..

[38] Yamada A., Kasahara K., Ogawa Y., Samejima K., Eriguchi M., Yano H., Mikasa K., Tsuruya K. (2019). Peritonitis Due to *Moraxella osloensis*: A Case Report and Literature Review. J. Infect. Chemother..

[39] Lee M., Kim M., Lee W., Kang S. (2021). Bacteremia Caused by *Moraxella osloensis*: A Fatal Case of an Immunocompromised Patient and Literature Review. Clin. Lab..

[40] Tabbuso T., Defourny L., Lali S.E., Pasdermadjian S., Gilliaux O. (2021). *Moraxella osloensis* Infection among Adults and Children: A Pediatric Case and Literature Review. Arch. Pédiatrie.

[41] Li Z., Xia J., Jiang L., Tan Y., An Y., Zhu X., Ruan J., Chen Z., Zhen H., Ma Y. (2021). Characterization of the Human Skin Resistome and Identification of Two Microbiota Cutotypes. Microbiome.

[42] Ide K., Saeki T., Arikawa K., Yoda T., Endoh T., Matsuhashi A., Takeyama H., Hosokawa M. (2022). Exploring Strain Diversity of Dominant Human Skin Bacterial Species Using Single-Cell Genome Sequencing. Front. Microbiol..

[43] Xia J., Li Z., Zhong Q., Wei Q., Jiang L., Duan C., Jia H., Tan Y., Han L., Wang J. (2023). Integration of Skin Phenome and Microbiome Reveals the key Role of Bacteria in Human Skin Agin. Res. Sq..

[44] Işeri L., Apan T., Şahin E. (2015). The Antimicrobial Susceptibility of Moraxella Species Other Than Moraxella Catarrhalis. Turk. Klin. J. Med. Sci..

[45] Ganzorig M., Lim J.Y., Hwang I., Lee K. (2018). Complete Genome Sequence of Multidrug-Resistant *Moraxella osloensis* NP7 with Multiple Plasmids Isolated from Human Skin. Microbiol. Soc. Korea.

[46] Hadi Ghaffoori Kanaan M., Riyadh Al-abodi H., Saad Abdullah S., Memariani M., Kohansal M., Ghasemian A. (2024). Biofilm Formation and Drug Resistance Determinants of Moraxella Catarrhalis, *Moraxella osloensis* and Moraxella Lacunata from Clinical Samples in Iraq. Health Biotechnol. Biopharma (HBB).

[47] Wagner C., Hänsch G.M. (2015). Pathophysiologie der implantatassoziierten Infektion. Orthopäde.

[48] Augustyniak D., Seredyński R., McClean S., Roszkowiak J., Roszniowski B., Smith D.L., Drulis-Kawa Z., Mackiewicz P. (2018). Virulence Factors of Moraxella Catarrhalis Outer Membrane Vesicles Are Major Targets for Cross-Reactive Antibodies and Have Adapted during Evolution. Sci. Rep..

